# Liquid Biopsy in Colorectal Cancer: Future Perspectives Through the Lens of Artificial Intelligence—A Comprehensive Review of Novel Literature

**DOI:** 10.3390/ijms27093951

**Published:** 2026-04-29

**Authors:** Dan Nicolae Paduraru, Alexandru Cosmin Palcău, Gabriel-Petre Gorecki, Alexandru Dinulescu, Maria-Luiza Băean

**Affiliations:** 1 Faculty of Medicine, Carol Davila University of Medicine and Pharmacy, 050474 Bucharest, Romania; dan.paduraru@umfcd.ro (D.N.P.); alexandru.dinulescu@drd.umfcd.ro (A.D.); 2 Surgery Department, CF2 Clinical Hospital, 011464 Bucharest, Romania; 3 Faculty of Medicine, Titu Maiorescu University, 031593 Bucharest, Romania; gabriel.gorecki@prof.utm.ro; 4 National Institute for Research and Development in Microtechnologies, 077190 Voluntari, Romania; 5 Department of Anesthesia and Intensive Care, CF2 Clinical Hospital, 011464 Bucharest, Romania; 6 Department of Pediatrics, Emergency Hospital for Children “Grigore Alexandrescu”, 011743 Bucharest, Romania; 7 Department of Fundamental Sciences, Faculty of Midwifery and Nursing, University of Medicine and Pharmacy “Carol Davila”, 050474 Bucharest, Romania; maria-luiza.baean@umfcd.ro; 8 Department of Clinical Laboratory of Radiology and Medical Imaging, CF2 Clinical Hospital, 011464 Bucharest, Romania

**Keywords:** liquid biopsy, colorectal cancer, artificial intelligence, machine learning, deep learning, fragmentomics, multi-cancer early detection

## Abstract

Colorectal cancer (CRC) remains one of the leading causes of cancer-related mortality worldwide, with prognosis critically dependent on the stage at diagnosis. Traditional tissue biopsy presents well-known limitations, including tumor heterogeneity and invasiveness. Liquid biopsy, encompassing the analysis of circulating tumor DNA (ctDNA), circulating tumor cells (CTCs), exosomes, and other cell-free biomarkers, has emerged as a transformative approach for non-invasive tumor profiling. This comprehensive narrative review outlines the recent evidence published on the current state and future perspectives of liquid biopsy in CRC, with a focused emphasis on the role of artificial intelligence (AI), machine learning (ML), and deep learning (DL) in data analysis and clinical translation. Methods: A narrative review of the literature was conducted by searching PubMed/MEDLINE, EMBASE, and ClinicalTrials.gov for articles published between January 2020 and January 2026, using a predefined Boolean search string combining terms related to liquid biopsy biomarkers, colorectal cancer, and artificial intelligence methodologies. Filters were applied to include only English-language human studies. Additional relevant sources were consulted to ensure comprehensive coverage of the available literature. Liquid biopsy platforms, particularly ctDNA sequencing and methylation profiling, demonstrate increasing clinical utility across the CRC care continuum from population screening to post-surgical minimal residual disease (MRD) detection and real-time therapy monitoring. AI-driven analytical frameworks, including Random Forest, Convolutional Neural Networks, LSTM models, and more recently Large Language Models (LLMs), substantially augment the sensitivity and specificity of liquid biopsy interpretation, enabling multimodal data integration. The convergence of liquid biopsy technology and AI-driven analytics represents a paradigm shift toward precision oncology in CRC. Remaining challenges include analytical standardization, model explainability, regulatory harmonization, and equitable access. Future integration of federated learning frameworks and LLM-based clinical decision support tools will be essential for responsible clinical translation.

## 1. Introduction

Colorectal cancer (CRC) is the third most commonly diagnosed malignancy and the second leading cause of cancer-related death globally, accounting for approximately 1.9 million new cases and 930,000 deaths annually according to the latest GLOBOCAN estimates [1]. Despite advances in surgical technique, systemic chemotherapy, and targeted biological agents, long-term outcomes remain heavily stage-dependent. While five-year survival exceeds 91% in localized disease, it falls to less than 14% in metastatic CRC [2]. This stark prognostic gradient underlines the critical importance of early and accurate disease detection, post-treatment surveillance, and real-time monitoring of therapeutic responses.

The current diagnostic standard (tissue biopsy followed by histopathological and molecular analysis), while foundational, suffers from inherent limitations. Intratumoral and intertumoral heterogeneity means that a single tissue sample captures only a spatial and temporal snapshot of tumor biology. This can potentially alter the broader genomic landscape reality [3]. Biopsy is also invasive, not always feasible due to tumor location, and poorly suited for serial monitoring over time [4]. These limitations are especially consequential in the era of precision oncology, where treatment decisions increasingly hinge on tumor mutational profiles, resistance mechanisms, and dynamic molecular changes. Unlike malignancies in anatomically inaccessible sites, such as lung or pancreatic cancer, where repeat tissue sampling carries significant procedural risk, colorectal tumors are relatively accessible via colonoscopy. The primary advantage of liquid biopsy in CRC therefore lies not in replacing tissue diagnosis but in enabling minimally invasive, serial longitudinal sampling that captures real-time tumor evolution, clonal dynamics, and emerging resistance mechanisms in a manner that repeat colonoscopic biopsy cannot practically achieve.

Against this backdrop, liquid biopsy has emerged as one of the most promising innovations in oncological diagnostics. Defined broadly as the analysis of tumor-derived material circulating in body fluids, most commonly peripheral blood, liquid biopsy provides a minimally invasive approach into tumor biology that is accessible, repeatable, and capable of capturing systemic tumor heterogeneity [5]. The main analytes of interest include circulating tumor DNA (ctDNA) and cell-free DNA (cfDNA), circulating tumor cells (CTCs), extracellular vesicles (EVs) and exosomes, circulating microRNAs (miRNAs), tumor-educated platelets (TEPs), and cell-free RNA [6,7]. Each analyte provides complementary biological information: ctDNA reflects the somatic mutational and epigenetic landscape of the tumor, CTCs enable single-cell genomic and phenotypic characterization, TEPs integrate tumor-derived RNA signals through a splicing-based mechanism, and exosomes carry proteomic and nucleic acid cargo reflective of the tumor microenvironment.

In non-small cell lung cancer (NSCLC), ctDNA-based detection of EGFR sensitizing and resistance mutations (T790M, C797S) is guideline-endorsed and has replaced repeat tissue biopsy in routine clinical practice for therapy selection and monitoring [8]. In breast cancer, ctDNA analysis has identified ESR1 and PIK3CA mutations as driving resistance to endocrine therapy and CDK4/6 inhibitors, with prospective trials demonstrating that ctDNA-guided treatment adaptation improves progression-free survival. In hepatocellular carcinoma, methylation-based cfDNA profiling enables early detection with high specificity in surveillance populations [9]. The CCGA (Circulating Cell-free Genome Atlas) program has validated multi-cancer early detection (MCED) based on cell-free methylation profiling across more than 50 cancer types, demonstrating the pan-oncological applicability of liquid biopsy platforms. These precedents provide critical context for the application and clinical translation of liquid biopsy in CRC.

A pivotal technological transition now reshaping liquid biopsy is the integration of artificial intelligence (AI) and its subfields, such as machine learning (ML) and deep learning (DL), into analytical pipelines [10]. AI refers broadly to computational systems designed to perform tasks that would otherwise require human intelligence, including pattern recognition, decision making, and predictive modeling. The volume and complexity of data generated by next-generation sequencing (NGS), methylation arrays, and proteomics platforms render traditional statistical approaches insufficient for pattern recognition at scale [11,12]. Classical ML approaches operate on structured, hand-crafted feature sets and have been extensively applied to clinical and genomic data for classification and survival prediction tasks. DL is a subclass of ML that employs artificial neural networks with multiple hierarchical layers of interconnected nodes, enabling the autonomous extraction of complex, high-dimensional features directly from raw data such as nucleotide sequences, fragment length distributions, or methylation profiles. AI-driven frameworks offer the capacity to identify subtle, non-linear biomarker signatures, integrate heterogeneous data modalities, and generate probabilistic clinical predictions with levels of granularity that surpass conventional methods [13]. From Random Forest classifiers predicting recurrence from ctDNA mutant allele fractions to Convolutional Neural Networks (CNNs) decoding nucleosome-positioning patterns in cfDNA and Large Language Models (LLMs) interpreting complex genomic reports in natural language, the landscape of AI in liquid biopsy is both rapidly evolving and clinically transformative. CNNs are particularly suited to spatially structured data, while recurrent architectures and transformers capture sequential and long-range dependencies, making them appropriate for longitudinal ctDNA dynamics and whole-genome cfDNA signals [14,15,16,17].

In the context of liquid biopsy, ML and DL algorithms are applied across a spectrum of tasks: classification of cancer versus non-cancer cfDNA signals, tissue-of-origin prediction, variant calling with error suppression, longitudinal tumor burden estimation, and integration of multiomic biomarker panels. The intersection of these computational approaches with liquid biopsy biomarkers represents a rapidly evolving paradigm with significant implications for early detection, minimal residual disease monitoring, and treatment response assessment in colorectal cancer.

This comprehensive narrative review aims to synthesize the current state of evidence on liquid biopsy in CRC, with particular emphasis on the spectrum of circulating biomarkers and their clinical utility, AI/ML/DL methodologies applied to liquid biopsy data, key clinical trials, and regulatory developments.

## 2. Methods

A narrative review of the literature was conducted. PubMed/MEDLINE, EMBASE, and ClinicalTrials.gov were searched for articles published between January 2020 and January 2026. The database search, article screening, and selection process were conducted iteratively between January and March 2026.The following Boolean search string was applied: (“liquid biopsy” OR “circulating tumor DNA” OR “ctDNA” OR “cell-free DNA” OR “cfDNA” OR “circulating tumor cells” OR “CTCs” OR “exosomes” OR “extracellular vesicles” OR “circulating miRNA”) AND (“colorectal cancer” OR “colorectal carcinoma” OR “colon cancer” OR “rectal cancer” OR “CRC”) AND (“machine learning” OR “deep learning” OR “artificial intelligence” OR “neural network” OR “random forest” OR “convolutional neural network” OR “large language model” OR “federated learning” OR “fragmentomics” OR “methylation profiling” OR “multi-cancer early detection”). Additional filters included English language and human studies. In addition to the articles identified through the initial search strategy, other relevant sources were explored to ensure a comprehensive coverage of the available literature. The search strategy was conducted in accordance with the specific requirements and indexing criteria of each database platform.

## 3. Results and Discussions

### 3.1. Biomarkers in Liquid Biopsy for Colorectal Cancer

The analytical resources of liquid biopsy in CRC encompass multiple distinct biomarker classes, each characterized by unique biological origins, detection platforms, and clinical utilities (Table 1).

It is important to distinguish between two conceptually distinct clinical applications of ctDNA in CRC. Early detection and screening aim to identify localized disease in asymptomatic individuals prior to clinical presentation. Post-treatment surveillance and therapeutic monitoring assess molecular residual disease and treatment response in patients with established diagnoses. While both applications leverage the same analytical platforms, they differ fundamentally in their clinical objectives, target populations, performance requirements, and regulatory pathways.

#### 3.1.1. Circulating Tumor DNA and Cell-Free DNA

ctDNA, defined as fragments of tumor-derived DNA shed into the bloodstream through apoptosis, necrosis, and active secretion, is the most extensively studied liquid biopsy analyte in CRC [18]. ctDNA harbors tumor-specific somatic mutations, copy number alterations (CNAs), and epigenetic modifications that reflect the molecular landscape of the originating tumor. Detection methodologies range from highly sensitive digital droplet PCR (ddPCR) for predefined hotspot mutations (KRAS, NRAS, BRAF, and PIK3CA) [19] to broad next-generation sequencing (NGS) panels capable of simultaneous multi-gene profiling and, more recently, to ultra-low-pass whole-genome sequencing (ULP-WGS) leveraged for fragmentomics analysis [20,21,22].

Clinical sensitivity is critically stage-dependent: studies report ctDNA detectability of 10–50% in Stage I CRC, 20–89% for Stage II, and 30–90% for Stage III, rising to over 90% in metastatic disease [23]. The DYNAMIC randomized controlled trial provided landmark evidence that ctDNA-guided adjuvant chemotherapy decisions in Stage II CRC were non-inferior to standard pathological risk stratification, with significant reductions in chemotherapy use without compromising recurrence-free survival [24,25]. Serial post-operative ctDNA monitoring has been shown to enable recurrence detection significantly ahead of clinical or radiological evidence. In a tumor-informed WGS-based cohort of stage III CRC patients, ctDNA positivity preceded radiological recurrence by a median of 8.7 months [26]. Complementary data from the BESPOKE CRC prospective observational study and the INTERCEPT study confirmed this lead time advantage and demonstrated that early molecular detection translates into higher rates of curative-intent local therapy [27,28,29].

Beyond mutation detection, the epigenetic landscape of ctDNA—particularly differential methylation at cancer-specific CpG loci—has become a major focus [30]. Genes such as SEPT9 (Septin 9), SDC2, VIM, and NDRG4 are aberrantly methylated in CRC tissue and detectable in cfDNA with high specificity, forming the analytical basis of approved non-invasive screening tests [31,32,33].

Emerging evidence suggests a biologically plausible and clinically relevant association between ctDNA and the “watch and wait” (W&W) strategy in rectal cancer. Post-neoadjuvant ctDNA clearance has been associated with a higher likelihood of complete pathological response and a lower risk of recurrence, supporting its potential role in identifying candidates suitable for non-operative management [34,35]. However, current data remain heterogeneous, and ctDNA lacks sufficient sensitivity and standardization.

#### 3.1.2. Circulating Tumor Cells

CTCs are intact, viable cells shed from primary or metastatic tumor deposits into the bloodstream. Unlike ctDNA, which provides molecular-genetic information, CTCs enable direct phenotypic, proteomic, and functional characterization of malignant cells, including single-cell genomic profiling [36]. The CellSearch platform remains, as of early 2026, the only FDA-cleared CTC enumeration system. However, next-generation microfluidic and label-free isolation technologies have substantially improved capture efficiency and downstream analytical resolution [37]. In CRC, CTC count has been correlated with overall survival in metastatic disease and may provide predictive information regarding response to anti-EGFR therapies through characterization of KRAS/NRAS mutational status [38,39,40]. Deep learning-based image classification systems have been deployed to automate CTC identification and subtype classification, reducing inter-observer variability and enabling high-throughput analysis [41,42].

#### 3.1.3. Exosomes, Extracellular Vesicles, and Tumor-Educated Platelets

Exosomes (30–150 nm diameter) and larger microvesicles are membrane-bound structures released by all cell types, including tumor cells. They carry a molecular cargo—proteins, lipids, mRNAs, miRNAs, and DNA—reflective of their cell of origin and play active roles in intercellular communication, immune modulation, and the preparation of pre-metastatic niches [43]. In CRC, tumor-derived exosomes have been shown to carry oncogenic cargo including KRAS-mutant DNA and immunosuppressive proteins, making them informative biomarkers for disease staging and drug resistance profiling [44,45].

Tumor-educated platelets (TEPs) represent a less conventional but increasingly studied liquid biopsy compartment. Upon contact with tumor-derived signals, circulating platelets undergo specific patterns of RNA splicing, creating tumor-educated transcriptomic profiles detectable by RNA sequencing [46]. SVM (support vector machine) algorithm and ensemble machine learning classifiers trained with TEP spliced RNA profiles have demonstrated promising pan-cancer diagnostic accuracy across multiple studies. The foundational work by Best et al. established a 96% overall accuracy across six tumor types, including CRC, using an SVM classifier [47]. The landmark large-scale validation by In’t Veld et al., encompassing 1096 blood samples from 18 cancer types, confirmed cancer detection in two-thirds of Stage I–IV patients at 99% specificity in asymptomatic controls, with tumor site-of-origin localization exceeding 80% accuracy [48]. More recently, interpretable ML frameworks incorporating neural networks and XGBoost applied to 2018 TEP RNA samples achieved AUC ~0.93 with SHAP-based feature attribution [49]. However, independent external validation has yielded substantially lower performance in disease-specific cohorts (AUC 0.54–0.55 in breast cancer) [50], underscoring that prospective, multi-institutional validation of TEP-based classifiers in CRC-specific populations remains an unmet need.

#### 3.1.4. Circulating microRNAs

miRNAs are small, non-coding RNAs that regulate gene expression post-transcriptionally. Tumor cells release miRNAs packaged in exosomes or bound to proteins into the circulation, where they exhibit remarkable stability [51]. In CRC, panels including miR-21, miR-92a, miR-141, miR-17-92 cluster, and miR-29a, have been proposed as diagnostic and prognostic biomarkers [52,53]. Machine learning classifiers applied to circulating miRNA panels in CRC have reported highly variable diagnostic performance across studies, reflecting differences in cohort composition, miRNA selection methodology, and platform. In case-control settings, panel-based ML approaches have yielded accuracies ranging from 0.868 to 0.947, with individual studies reporting sensitivity/specificity pairs of 93.8%/91.3% for a three-miRNA panel (miR-144-3p, miR-425-5p, and miR-1260b) [54,55] and 0.895 accuracy for a seven-miRNA signature [56]. However, the absence of large-scale, prospectively validated, multi-institutional ML-miRNA studies in CRC prevents definitive performance benchmarking, and cross-study comparisons are substantially limited by pre-analytical and cohort heterogeneity.

### 3.2. Technical Platforms and Analytical Standards

The clinical utility of liquid biopsy is inseparable from the analytical platforms underpinning biomarker detection. Two dominant sequencing paradigms govern ctDNA analysis: digital droplet PCR (ddPCR) and next-generation sequencing (NGS). Digital droplet PCR offers high analytical sensitivity for predefined hotspot mutations, with reported limits of detection in the range of 0.01–0.1% mutant allele fraction under clinical conditions—substantially superior to the 1% threshold of standard NGS-based quantification and the 0.5% of conventional qPCR approaches. This sensitivity renders ddPCR well-suited for monitoring known driver mutations in CRC, including KRAS G12V/D, NRAS, and BRAF V600E, in plasma cfDNA, where tumor-derived mutant alleles are often present at very low fractional abundances, particularly in non-metastatic and post-operative settings [57,58].

NGS-based approaches, encompassing targeted gene panels, hybrid-capture whole-exome sequencing, and low-pass WGS, allow simultaneous interrogation of hundreds of cancer-relevant genes and genome-wide copy number landscapes [59]. The introduction of error-suppression strategies like unique molecular identifiers (UMIs), duplex sequencing, and background polishing algorithms has driven sequencing noise to levels permitting reliable ctDNA detection at allele fractions below 0.1% [60]. Methylation profiling, central to Multi-Cancer Early Detection (MCED) platforms such as Galleri (Grail), employs targeted or genome-wide bisulfite sequencing to identify tissue-specific methylation signatures in cfDNA [61].

Despite this technological sophistication, pre-analytical variables exert profound influences on liquid biopsy results. Blood collection tube type (EDTA versus cell-stabilizing Streck tubes), time to processing, plasma isolation methodology, cfDNA extraction kit selection, and storage conditions all introduce variability. Harmonization efforts led by the BLOODPAC consortium and the European Liquid Biopsy Society (ELBS) have produced consensus pre-analytical guidelines, yet full standardization across clinical laboratories remains elusive. This variability is particularly consequential for AI training datasets. Batch effects and pre-analytical disturbances can substantially confound model performance and generalizability [62,63].

### 3.3. Artificial Intelligence, Machine Learning, and Deep Learning in Liquid Biopsy for CRC

The intersection of liquid biopsy and AI represents perhaps the most consequential frontier in contemporary oncology diagnostics. The multi-dimensional, high-noise nature of liquid biopsy data—encompassing somatic variant calls, copy number profiles, methylation matrices, and fragmentation landscapes—creates precisely the analytical environment in which AI-based methods excel.

#### 3.3.1. Why AI Is Necessary: The Data Problem in Liquid Biopsy

Traditional statistical methods—logistic regression and Cox proportional hazards models—assume linear relationships between predictors and outcomes and struggle with high-dimensional, correlated feature spaces. Liquid biopsy generates data with dimensionality that routinely exceeds available sample sizes: a single cfDNA WGS experiment may yield millions of fragment-level measurements across the genome [64], while methylation arrays interrogate over 850,000 CpG sites simultaneously [65]. Furthermore, the biological signal is inherently heterogeneous, reflecting clonal dynamics, tumor fraction variability, and competing non-malignant DNA sources (e.g., clonal hematopoiesis of indeterminate potential—CHIP) [66]. AI models, particularly ensemble methods and deep neural networks, are uniquely positioned to capture complex, non-linear relationships across these data spaces, reduce false positive rates from germline and CHIP variants, and integrate orthogonal biological information simultaneously [67,68].

#### 3.3.2. Classical Machine Learning: Ensemble Methods and Feature Engineering

Among classical ML approaches, Random Forest and gradient-boosted tree algorithms (XGBoost, LightGBM) have emerged as workhorses for ctDNA-based classification tasks in CRC [69,70]. ML classifiers applied to ctDNA-derived features such as mutant allele fraction and variant allele frequency have been explored for recurrence prediction in CRC, with tree-based models on clinicopathological and molecular data reporting AUC values broadly in the range of 0.80–0.99 across retrospective cohorts [71,72,73].

Support Vector Machine (SVM) classifiers have been applied to multiple cfDNA-derived feature types for CRC-versus-healthy discrimination. In a large cohort of 546 CRC patients and 271 non-cancer controls, an ML approach incorporating SVM on whole-genome cfDNA sequencing data—capturing gene body read count patterns reflective of both tumor and non-tumor contributions—achieved a mean AUC of 0.92 and sensitivity of 85% at 85% specificity, with performance highest in early-stage (Stage I/II) disease [74]. SVM classifiers have additionally been applied to cfDNA methylation marker panels in CRC tissue and plasma, with SEPT9/AKR1B1 methylation-based SVM models reporting 98% sensitivity and 99% specificity in tissue-based case-control settings [75], though plasma-based external validation of these methylation-SVM models at scale remains pending. The application of SVM specifically to circulating miRNA panels in liquid biopsy for CRC is supported by exploratory studies but lacks large-scale prospective validation [76]. However, SVMs require careful hyperparameter tuning and struggle with datasets of moderate size (*n* < 500), limiting their scalability to multi-institutional real-world settings. Regularized regression methods—LASSO, ElasticNet, etc.—have been employed for biomarker panel optimization, identifying minimal, clinically actionable miRNA or methylation signatures from larger candidate pools [77,78].

#### 3.3.3. Deep Learning: Neural Architectures for Liquid Biopsy Data

Deep learning introduces hierarchical feature extraction that bypasses the manual feature engineering requirement of classical ML. Convolutional Neural Networks (CNNs) have been applied to two distinct liquid biopsy domains in CRC. First, in CTC analysis, CNNs trained on CellSearch immunofluorescence images learn discriminative morphological and immunophenotypic features that outperform rule-based CTC classifiers, with human-expert-level accuracy achieved for CTC/WBC discrimination [79]. Second, and more profoundly, CNNs have been adapted for one-dimensional “image-like” representations of cfDNA fragmentation patterns (nucleosome-positioning scores, fragment length distributions, and chromatin accessibility inferences) [80], enabling tissue-of-origin attribution and early cancer detection in approaches pioneered by studies such as DELFI (DNA Evaluation of Fragments for Early Interception) [81].

Recurrent neural networks (RNNs) and Long Short-Term Memory (LSTM) architectures are particularly suited for sequential or longitudinal liquid biopsy data, for instance, modeling the trajectory of ctDNA levels across serial blood draws during chemotherapy to predict eventual pathological response or emerging resistance [82]. Transformer-based architectures, originally developed for natural language processing, are being adapted for genomic sequence modeling, offering potential for simultaneous analysis of mutation, methylation, and fragmentation features within a unified model [83,84].

Graph Neural Networks represent a conceptually promising architecture for future liquid biopsy applications in CRC, given their inherent capacity to model complex, non-linear relationships between molecular entities, such as integrating ctDNA-detected somatic variants with protein–protein interaction (PPI) network topology for drug resistance prediction. Current validated GNN applications in CRC remain confined to digital histopathology—including cell graph convolutional networks for tumor grading and spatial omics-based immune infiltration pattern recognition and to pan-cancer multi-omics subtype classification frameworks using tissue-derived data [85,86,87].

#### 3.3.4. Fragmentomics and AI

Fragmentomics represents the systematic analysis of cfDNA fragment length, end motifs, nucleosome positioning, and genomic coverage patterns. This innovation has emerged as one of the most information-rich dimensions of liquid biopsy, particularly enabled by AI [88]. The DELFI study demonstrated that genome-wide cfDNA fragmentation profiles captured by shallow WGS harbored tumor-specific signals detectable by ML classifiers even at very low ctDNA fractions (<1%), with AUC values of 0.94 for cancer detection across multiple cancer types, including CRC [81]. Subsequent studies have refined fragmentomics pipelines to specifically address CRC, incorporating preferred end motif (PEM) analysis, open chromatin inference, and transcription factor footprinting from cfDNA [89].

AI models applied to fragmentomics data must grapple with confounding factors including age-related cfDNA fragmentation patterns, inflammatory conditions, and clonal hematopoiesis [90]. Multivariable deep learning models that simultaneously account for these covariates, alongside tumor-specific signals, have demonstrated substantially reduced false-positive rates compared to single-feature approaches [91,92]. Importantly, fragmentomics is largely mutation-agnostic, enabling cancer detection in tumors without common driver mutations and offering orthogonal information to mutation-based ctDNA analysis [93,94].

#### 3.3.5. Methylation Profiling, MCED, and AI

The methylome of circulating cfDNA provides one of the richest and most tissue-specific information layers accessible through liquid biopsy. Cancer cells undergo extensive epigenomic reprogramming, resulting in characteristic patterns of aberrant CpG methylation that are preserved in cfDNA shed into the blood [95]. Grail’s Galleri assay—the most advanced commercially available multi-cancer early detection (MCED) test—employs targeted methylation sequencing covering more than 100,000 genomic regions encompassing over 1.1 million CpG sites, analyzed by machine learning classifiers to simultaneously detect a cancer signal and predict tissue of origin across more than 50 cancer types [96]. In the foundational CCGA3 clinical validation study comprising 4077 participants in an independent validation set, the MCED test demonstrated an overall sensitivity of 51.5% across all cancer types at 99.5% specificity, with cancer signal origin (CSO) prediction accuracy of 88.7%. For the 12 pre-specified cancers responsible for approximately two-thirds of annual U.S. cancer deaths—including colorectal cancer—Stage I–III sensitivity reached 67.6% [97]. The prospective PATHFINDER cohort study evaluated the refined clinical version of Galleri in an asymptomatic screening population, reporting a specificity of 99.1%, PPV (positive predicting value) of 43.1%, and CSO accuracy of 85%, with a cancer signal detected in 1.4% of participants; sensitivity in this prospective screening context was substantially lower than in case-controlled validation, consistent with the lower cancer prevalence in an asymptomatic population [98]. It is important to contextualize these metrics within their clinical implications. In a screening population with low cancer prevalence, a PPV of 43.1% indicates that the majority of screen-positive individuals will not have an underlying malignancy, necessitating extensive and potentially invasive diagnostic workup. This has significant implications for patient anxiety, healthcare resource allocation, and the risk of overdiagnosis. These considerations must be weighed against the potential benefit of earlier cancer detection when evaluating the population-level implementation of MCED testing.

The computational architecture underlying Galleri has been described in the analytical validation literature as comprising two sequential machine learning components: an ensemble logistic regression classifier applied to source models of cancer-specific methylation patterns from the targeted bisulfite sequencing data to generate a cancer detection score, followed by a separate cancer signal origin (CSO) classifier applied to tissue-specific methylation source models to predict the anatomic location of the primary tumor. The detection classifier assigns scores relative to the 99.4th percentile threshold of non-cancer training samples, providing a binary cancer/non-cancer call at a fixed high-specificity operating point [97,99]. It should be noted that other MCED platforms differ substantially in their underlying technology. For example, CancerSEEK integrates ctDNA mutational analysis with serum protein biomarkers using logistic regression classifiers [100].

CRC-specific methylation markers like SEPT9, SDC2, NDRG4, and VIM have been incorporated into clinically deployed assays with well-characterized performance [32]. ColoSense™ (Geneoscopy, Inc., St. Louis, MO, USA), a multi-target stool RNA test detecting eight colorectal neoplasia-associated RNA transcripts alongside occult hemoglobin, received FDA approval in May 2024 for average-risk adults aged 45 and older. The pivotal phase III CRC-PREVENT trial (NCT04739722) demonstrated 94% sensitivity for CRC, including 100% sensitivity for Stage I disease, and 46% sensitivity for advanced adenomas [101]. In parallel, Exact Sciences’ next-generation multitarget stool DNA test, Cologuard Plus™, received FDA approval in October 2024 based on the BLUE-C pivotal study (NCT04144738), reporting 93.9% sensitivity for CRC and 43.4% sensitivity for advanced precancerous lesions [102].

#### 3.3.6. Large Language Models in Liquid Biopsy Interpretation

The recent emergence of LLM transformer-based neural networks trained on vast biomedical corpora and on multimodal biological data including genomic sequences introduces a qualitatively new capability into precision oncology: the automated generation and interpretation of natural language from complex genomic reports and clinical contexts [103]. Foundational studies have demonstrated that conversational LLMs (including ChatGPT version 3, Galactica, and BioMedLM) can identify relevant treatment options from somatic mutation profiles in oncology cases, though performance currently deviates substantially from expert molecular tumor board recommendations and is not yet applicable as a routine clinical decision-support tool [104]. In parallel, domain-adapted genomic LLMs—trained on nucleotide sequences using transformer architectures—are extending natural language modeling paradigms to raw biological sequence interpretation, opening new analytical frontiers in cfDNA and epigenomic data analysis [105].

In the specific context of liquid biopsy, LLMs are being explored as clinical decision support tools that integrate ctDNA findings with patient history, prior treatment lines, imaging results, and established therapeutic guidelines (ESMO/NCCN) to generate actionable recommendations. A key advantage is the ability to synthesize heterogeneous information sources into coherent, clinically relevant outputs in real time [106]. However, LLMs are prone to “hallucination” (generation of plausible but factually incorrect statements), lack transparent reasoning, and have not yet been validated in prospective clinical liquid biopsy workflows [107,108]. Responsible deployment will require rigorous accuracy benchmarking against expert molecular tumor boards, model uncertainty quantification, and robust human oversight frameworks.

#### 3.3.7. Explainability and Clinical Trust (XAI)

A fundamental problem in clinical use of AI is the distance between model complexity and interpretability. Ensemble deep learning models that integrate fragmentomics, methylation, and mutation data may achieve outstanding predictive accuracy but are hard for clinical interpretation. Clinicians, patients, and regulators require insight into the reasoning underpinning an AI-generated diagnosis or treatment recommendation, particularly in high-stakes oncological decisions.

The field of explainable AI (XAI) has developed multiple frameworks to address this challenge. SHAP (SHapley Additive exPlanations) values decompose a model’s output into per-feature contributions, enabling attribution of a ctDNA-based recurrence risk score to specific genomic alterations or fragmentation features [109,110]. LIME (Local Interpretable Model-Agnostic Explanations) approximates complex model decisions locally with simpler interpretable surrogates [111,112]. Attention mechanisms in transformer architectures provide alignment-based explanations highlighting which genomic regions drove a particular classification. Regulatory bodies including the FDA and EMA are increasingly requiring XAI documentation as part of AI/ML-based IVD submissions, making explainability not merely a scientific desideratum but a regulatory necessity.

### 3.4. Clinical Applications: Current Evidence and Ongoing Studies

The clinical translation of liquid biopsy in CRC has progressed from exploratory biomarker studies to prospective randomized controlled trials across the disease continuum (Table 2).

#### 3.4.1. Population Screening

The CRC screening landscape has been transformed by next-generation liquid biopsy-based tests. The Epi proColon test (SEPT9 methylation in plasma) was the first blood-based CRC screening test approved by the FDA in 2016 [118]. More recently, the ColoSense test’s approval in 2024 [119] and the ongoing PATHFINDER 2 study evaluating Galleri in routine clinical practice mark significant milestones [120]. The fundamental advantage of blood-based screening over colonoscopy—higher acceptability, accessibility, and absence of bowel preparation—is particularly relevant in populations with low colonoscopy compliance [121]. AI-driven risk stratification algorithms that combine ctDNA results with clinical risk factors (age, BMI, family history, and microbiome composition) are under development to further refine screening precision and reduce unnecessary colonoscopy referrals [122,123].

#### 3.4.2. Minimal Residual Disease Detection and Adjuvant Therapy Guidance

Post-surgical MRD detection represents the clinical application with perhaps the most compelling prospective evidence for ctDNA in CRC. Multiple studies have demonstrated that ctDNA positivity in the 4–6 weeks post-resection is the strongest prognostic biomarker for recurrence, independent of pathological stage. The DYNAMIC RCT established that ctDNA-guided omission of adjuvant chemotherapy in ctDNA-negative Stage II CRC patients was safe and reduced chemotherapy overtreatment [25]. CIRCULATE-Japan is evaluating ctDNA and the risk of recurrence in ctDNA-positive patients, while the BESPOKE CRC cohort has documented that serial ctDNA monitoring detects molecular recurrence before clinical relapse [27,114,115]. Serial ctDNA monitoring has been shown to detect molecular recurrence a median of 8.7 months before clinical relapse in Stage III CRC [26]. AI models trained on serial ctDNA add prognostic granularity beyond simple ctDNA positivity/negativity, identifying patients at highest risk for early systemic relapse [124].

#### 3.4.3. Monitoring Therapeutic Response and Detecting Acquired Resistance

In metastatic CRC, ctDNA dynamics correlate with radiological response to chemotherapy (FOLFOX and FOLFIRI) and targeted agents (bevacizumab, cetuximab, and panitumumab) [125]. Studies have demonstrated that early ctDNA decline predicts ultimate pathological and radiological response with greater temporal resolution than conventional cross-sectional imaging [126,127]. Critically, ctDNA analysis enables early identification of acquired resistance mutations—particularly KRAS/NRAS/BRAF and EGFR ectodomain mutations emerging under anti-EGFR selective pressure—weeks to months before radiological progression [127,128]. The concept of “rechallenge”, with anti-EGFR agents in patients whose ctDNA has cleared resistance mutations after EGFR-free intervals is supported by data from the CRICKET and CHRONOS trials [129,130,131].

## 4. Challenges and Limitations

Despite its considerable promise, the clinical adoption of AI-enhanced liquid biopsy in CRC faces multifactorial challenges spanning technical, clinical and socio-economic domains.

### 4.1. Technical and Biological Challenges

Sensitivity in early-stage CRC remains a fundamental limitation. The mutant allele fraction (MAF) of ctDNA in Stage I disease is typically 0.01–0.1%, approaching the error floor of most sequencing platforms and requiring ultra-deep sequencing (>10,000× coverage) to detect reliably [132,133]. Clonal hematopoiesis of indeterminate potential (CHIP) are somatic mutations in hematopoietic stem cells that are shared with solid tumor drivers, like TP53 and KRAS, as mentioned before. This is a major confounder in cfDNA analysis, particularly in older patients who constitute the highest CRC-risk group. Distinguishing CHIP-derived variants from true tumor variants requires paired white blood cell sequencing, adding cost and complexity [134,135]. Pre-analytical variability in sample handling, as detailed above, introduces batch effects that degrade AI model performance when models trained on one institutional cohort are deployed in another [136,137]. This is a critical obstacle to generalizability of protocols and results.

### 4.2. AI-Specific Limitations

AI models for liquid biopsy suffer from several characteristic failure modes. Dataset imbalance—reflecting the relative scarcity of early-stage cancer samples and the abundance of control and late-stage specimens—biases classifiers toward the majority class, inflating apparent AUC metrics without genuine clinical sensitivity improvement. Most published AI liquid biopsy studies use retrospective, often single-institution datasets that do not represent the ethnic, genetic, and clinical diversity of real-world CRC populations. Finally, the lack of prospective, randomized evidence that AI-guided liquid biopsy decisions improve patient outcomes (rather than merely biomarker performance metrics) remains a critical gap [138].

### 4.3. Cost and Accessibility

Commercial ctDNA assays costs around USD 3000 to USD 9000, currently limiting their use to well-resourced healthcare systems. Coverage for ctDNA-guided MRD monitoring in early-stage CRC is not yet established in most health systems globally. AI-enhanced MCED tests involve additional infrastructure costs for the ML analytics platform. The deployment of federated learning systems and LLM-based decision support tools requires substantial institutional computational infrastructure. Addressing cost and access barriers is essential to prevent liquid biopsy from becoming a technology that amplifies existing healthcare inequalities.

## 5. Conclusions

Liquid biopsy has evolved from a research curiosity to a clinically actionable diagnostic and monitoring tool in colorectal cancer. The evidence base is expanding rapidly across screening, minimal residual disease detection, treatment monitoring, and resistance profiling. The analytical complexity of multi-biomarker liquid biopsy data, encompassing ctDNA mutations, methylation patterns, fragmentation landscapes, CTCs, and exosomal cargo, makes the integration of AI not merely beneficial but necessary for maximizing clinical information yield.

Machine learning and deep learning models have demonstrated compelling performance across the CRC liquid biopsy spectrum: ensemble classifiers for recurrence prediction, CNNs for fragmentomics and CTC analysis, LSTM networks for longitudinal ctDNA trajectory modeling, GNNs for resistance network mapping, and LLMs for genomic report interpretation. Methylation-based multi-cancer early detection platforms represent a paradigm shift toward blood-based cancer screening. Federated learning frameworks offer a path toward large-scale, privacy-preserving AI model development that transcends institutional boundaries.

Substantial challenges remain. Sensitivity in early-stage disease, standardization of pre-analytical protocols, algorithmic bias and dataset diversity, regulatory harmonization for adaptive AI, and healthcare system cost barriers each demand focused research and policy attention. The ethical dimensions of AI-driven genomic diagnostics are not peripheral considerations but core requirements for responsible deployment.

Looking forward, the convergence of ultra-sensitive liquid biopsy platforms, multimodal AI, digital twin oncology models, and point-of-care sequencing technologies represents a future in which continuous, non-invasive molecular monitoring of colorectal cancer becomes a clinical reality. The evidence base reviewed here confirms that the trajectory is set; the imperative now is to ensure that the journey is guided by scientific rigor, clinical utility, and the equitable benefit of all patients at risk from this prevalent and preventable disease.

## Figures and Tables

**Table 1:** Overview of Liquid Biopsy Biomarkers in CRC: Characteristics and AI Integration.

Biomarker	Clinical Application	Key Platform	Sensitivity/Specificity	AI Integration
ctDNA/cfDNA	Screening, MRD, therapy monitoring, recurrence detection	ddPCR; NGS (WGS, targeted)	Stage I: 10–50%; Stage IV: >90%	CNN, Random Forest for variant calling; fragmentomics models
CTCs	Metastatic potential, drug resistance profiling	CellSearch; microfluidics	Moderate (stage-dependent); high specificity	Image-based DL (CNN) for CTC classification
Exosomes/EVs	Tumor microenvironment, drug resistance signals	NanoSight; SEC isolation	Emerging; variable	Graph Neural Networks; proteomics DL pipelines
miRNA circulating	Early detection panels (miR-21; miR-92a; miR-141)	qPCR; NGS	Panels: ~80–85% Sens/~80–90% Spec	Ensemble ML classifiers on miRNA panels
Methylated DNA (cfDNA)	Pan-cancer and CRC-specific screening (SEPT9; SDC2)	Bisulfite NGS; EPIC arrays	SEPT9: 70–90%/>90%; Galleri: ~60% overall early	Methylation ML models (tissue-of-origin; MCED)
TEPs (tumor-educated platelets)	Treatment response, metastasis prediction	RNA-seq	Emerging (~80% in select studies)	Random Forest on spliced RNA profiles

**Table 2:** Key Clinical Trials and Studies in Liquid Biopsy for CRC.

Trial/Study	Phase	Biomarker	Population	Key Finding
DYNAMIC [24,113]	II/III RCT	ctDNA	Stage II CRC	ctDNA-guided adjuvant chemo reduced overtreatment; ctDNA-neg patients safely avoided chemotherapy
CIRCULATE-Japan GALAXY [114]	Cohort + RCT	ctDNA	Stages II–IV	ctDNA positivity post-op strong predictor of recurrence; ctDNA-guided adjuvant arms ongoing
BESPOKE CRC [27,115]	Prospective Cohort	ctDNA	Stage II–III CRC	Serial ctDNA monitoring enabled early recurrence detection; landmark study for MRD in CRC
COBRA [116]	Phase II/III RCT	ctDNA (Guardant)	Stage II CRC	Evaluating ctDNA to guide adjuvant chemotherapy decisions; primary endpoint—NOT MET
PATHFINDER [98]	Prospective Cohort	Methylated cfDNA	General screening population	feasibility of MCED screening for cancer, including CRC;
HIBRID [117]	Methodologic study (trained on DACHS cohort + validated on GALAXY cohort)	ctDNA/MRD; DL applied on histology analysis—WSI (whole slide images)	Stage II–IV CRC	DL model on H&E WSIs combined with ctDNA-based MRD status improves prognostic stratification beyond ctDNA alone; MRD-negative/DL high-risk patients benefit from adjuvant chemotherapy, identifying a clinically actionable subpopulation invisible to liquid biopsy alone.

## Data Availability

No new data were created or analyzed in this study. Data sharing is not applicable to this article.
